# The research behind a taxonomic monograph: a case study from *Ipomoea* (Convolvulaceae)

**DOI:** 10.1007/s12225-024-10184-6

**Published:** 2024-11-21

**Authors:** Pablo Muñoz-Rodríguez, Tom Carruthers, Tom Wells, Alex Sumadijaya, John R. I. Wood, Robert W. Scotland

**Affiliations:** 1 Department of Biodiversity, Ecology and Evolution, Faculty of Life Sciences, Complutense University of Madrid, José Antonio Novais 12, 28040 Madrid, Spain; 2 Department of Biology, University of Oxford, South Parks Road, Oxford, OX1 3RB UK; 3 Department of Ecology and Evolutionary Biology, Biological Sciences Building, University of Michigan, 1105 North University Ave, Ann Arbor, MI 48109-1085 U.S.A.; 4 Royal Botanic Gardens, Kew, Richmond, TW9 3AE UK; 5 Herbarium Bogoriense, Research Center for Biosystematics and Evolution, National Research and Innovation Agency (BRIN), KST Sukarno, Cibinong, 16911 West Java Indonesia

**Keywords:** biodiversity, botany, global study, herbarium specimens, manuscript methodology, molecular data, systematics, tropical plants.

## Abstract

Systematic monographs are an important tool for understanding biodiversity. However, while papers that outline systematic methods for biogeography, phylogenetics and diversification are commonplace, papers that cover methods for monographic and taxonomic research are rare. In this paper, we describe how we conducted a monographic study of *Ipomoea*, drawing attention to the resources we made use of and the practical steps we took, with a particular focus on how we integrated results from molecular and morphological analyses. The monograph provided a framework for a range of subsequent research, including studies on the origin of the important crop sweet potato. It is hoped that our experience will provide a blueprint for others embarking on the preparation of a systematic monograph.

## Introduction

Systematic biology, or simply systematics, is the science that deals with the naming and description of organisms, their classification, their geographical distribution, and their evolutionary history (Michener *et al.* 1970). It aims to answer four main questions about an organism, each of them more or less pertaining to a different discipline: *what is it?* (Taxonomy); *what are its evolutionary relationships?* (Phylogenetics); *where does it live?* (Biogeography); and, increasingly, *how old is it?* (Divergence times). It is often the case, given the specific expertise required, that these four questions are dealt with independently with little or no interaction between disciplines.

Answering the first question — *What is it?* — is, in fact, an essential pre-requisite to accurately answering the other three questions. Unfortunately, due to lack of available expertise, funding constraints, and the time it takes to conduct taxonomic revisions, this initial question is often neglected. This has fundamental — although often overlooked — implications for the accuracy and comparability of studies addressing the other three questions (e.g. large-scale phylogenetic analyses, ecological studies). The reliability of the taxonomic knowledge is an essential foundation not only for groups in which existing knowledge is generally good, but also for those groups in which it is incomplete and/or strewn with error.

### Accelerating taxonomy

Over a decade ago, the two senior authors on this paper began to consider how to accelerate and improve the taxonomic study of poorly known tropical plant groups (Scotland & Wood 2012). They were concerned that monographs were excessively detailed and took many years — often the lifetime of the researcher — to complete, partly because they generally aim at comprehensiveness and perfection. For some groups, in particular large tropical plant groups, knowledge is so poor and fragmentary that producing a monograph in a reasonable time is simply not possible. This partially explains the small number of completed monographs published to date (Muñoz-Rodríguez *et al.* 2023a).

Given the stated power and importance of taxonomic monographs — i.e., the study of plant groups on a global scale — for wider biodiversity studies (Box 1), we proposed the ‘Foundation Monograph’ concept (Scotland & Wood 2012; Wood *et al.* 2015b; Muñoz-Rodríguez *et al.* 2019) as an alternative to the more *traditional* monograph. A foundation monograph seeks to prioritise and synthesise the most important elements in a taxonomic study — morphology, phylogeny, and geography — to revise a group across its entire distribution, in a reasonable time frame. In many species-rich groups of tropical plants, much progress can be achieved through a study of existing collections augmented by selective fieldwork and integrated use of morphological and molecular sequence data. Our aim is not to obtain a complete, perfect knowledge of every species in the group, but to provide a reliable taxonomic framework and lay the groundwork for subsequent studies. In other words, a foundation monograph is the global foundation on which a more detailed and local knowledge of the group can be more accurately built.

**Table:** 

**Box 1. Why taxonomic monographs are important**
A monograph captures all relevant existing taxonomic knowledge about a group of organisms at the time of publication, constituting the most up-to-date source of information for that group and providing the necessary taxonomic support for subsequent studies A monograph includes information on: - Nomenclature (including synonyms, types, notes) - Descriptions - General and detailed distribution - Basic information on habitat and ecology - Assessment of conservation status (in recent years, if available) - Reference specimens - Basic information on recorded uses (if available) - Cytology and micromorphology (if available) - Information on phylogenetic relationships (if available) To be most useful, a monograph should also include tools that allow other researchers and users to identify specimens, such as identification keys, photographs, maps, and illustrations

We first produced a Foundation Monograph of *Convolvulus* L. (Wood *et al.* 2015b), and then moved on to work on the much larger genus *Ipomoea* L. (Wood *et al.* 2020), building on what we had learnt. We have since continued to advocate the importance of botanical monographs, and foundation monographs in particular, emphasising the implications of this work beyond taxonomy (Williams *et al.* 2014; Wood *et al.* 2015b, 2020; Muñoz-Rodríguez *et al.* 2019, 2023a).

### How to write a taxonomic monograph

Although there are many papers advocating the importance and comprehensive nature of taxonomic monographs (e.g., Marhold *et al.* 2013; Vogel Ely *et al.* 2017; Gorneau *et al.* 2022), the methodology of producing a monograph has rarely been systematically explained (see, for example, Bremekamp 1970). In this paper we describe our method and its justification, so this paper can be seen as a report of ‘how we researched a taxonomic monograph’. We deliberately use the phrase ‘how we researched’ to make clear this is only one approach amongst a myriad of research options available for monographic research — but one that we have developed and implemented and shown that works. Although our approach is largely motivated by our work on *Ipomoea* and *Convolvulus*, it also benefits from our experience with other plant groups, such as *Acalypha* L. (Euphorbiaceae), *Jacquemontia* Choisy (Convolvulaceae), *Salvia* L. (Lamiaceae), *Strobilanthes* Blume (Acanthaceae), and *Tecoma* Juss. (Bignoniaceae).

Some of the contents of this paper were published as supplementary information to our monographic study of *Ipomoea* in Muñoz-Rodríguez *et al.* (2019) but are re-published here in a modified form. Specifically, here we focus on *what we did and why we did it*. In doing so, we attempt to provide a global view of our experiences which might be of value to other researchers. We therefore include a discussion of its impact on our own research and the consequent stimulation for wider research both by us and by other botanists working on *Ipomoea* post publication. Our aim is to provide a written record of how we conducted our monographic study, and to demonstrate that the impact of this work went far beyond the taxonomic monograph itself (Box 2).

**Table:** 

**Box 2. Impact of poor taxonomic knowledge**
Failing to correctly name and describe a specimen can have dramatic consequences. The number of herbaria has doubled in recent decades, and the number of herbarium specimens has grown exponentially (Bebber *et al.* 2010; Goodwin *et al.* 2015). At the same time, it has been estimated that more than 50% of all tropical plant herbarium specimens are likely to be incorrectly named (Goodwin *et al.* 2015). The exponential growth in data accumulation and the long-foreseen decline in taxonomic studies is likely to exacerbate this trend, with implications for other disciplines such as forest ecology and biodiversity conservation (Feeley 2015; Vogel Ely *et al.* 2017). As an example, in our study of American *Ipomoea* (Wood *et al.* 2015a, 2017, 2020; Muñoz-Rodríguez *et al.* 2019), 40% of nearly 2,000 specimens that we sequenced (and a similar number of those we did not sequence) required a different name as a result of our taxonomic revision (see Fig. 3b in Muñoz-Rodríguez *et al.* 2019). Similar values have been reported for other groups of tropical plants, where misidentified specimens are widespread (Goodwin *et al.* 2015). This demonstrates that taxonomic revisions are essential for poorly known plant groups, and especially species-rich groups such as *Ipomoea*, as otherwise the level of error incorporated in any biodiversity study using these specimens would be massive

We divide the paper into three sections: 1) Groundwork, 2) Taxonomic study & workflow, and 3) After publication: impact and new studies enabled by the monograph. All sections and subsections are illustrated with examples from our own work on *Ipomoea* and *Convolvulus*.

## GROUNDWORK

We started our work with a series of preparatory steps, essential to guarantee the successful completion of a monograph. These included the elaboration of a working list of plant names, a thorough bibliographic review, the establishment of contacts and correspondence with researchers in other relevant institutions and countries, and the selection of specimens to study.

### Preliminary checklist

A necessary first step to monographing a plant group is the compilation of a preliminary list of all relevant names in recent use. This provides a base line from which subsequent work can be developed.

As a starting point in the preparation of the monograph of *Ipomoea*, for example, we compiled a list of all names in recent use belonging to genera in the Convolvulaceae tribe *Ipomoeeae* Hallier f. (species with spiny pollen): *Ipomoea*, *Argyreia* Lour., *Astripomoea* A.Meeuse, *Blinkworthia* Choisy, *Lepistemon* Blume, *Lepistemonopsis* Dammer, *Rivea* Choisy, *Stictocardia* Hallier f. and *Turbina* Raf. These segregate genera had been shown to be nested within *Ipomoea* by previous authors (Manos *et al.* 2001; Stefanovic *et al.* 2002, 2003) and thus were also of interest.^1^ Our preliminary checklist contained some 800 names and was largely based on published literature (see complete list in Muñoz-Rodríguez *et al.* 2019). We complemented the checklist with the approximate geographical distribution of each species by major area, i.e., continental region or country.

During our work, we searched for synonyms and infraspecific taxa in different works. Choisy’s account of *Ipomoea* in De Candolle’s *Prodromus* (Choisy 1845) provided a comprehensive list of pre-1845 synonyms which we could use to trace back names published in the works of Don, Rafinesque, Vahl, Willdenow, Desrousseaux, Roemer and Schultes, Sprengel, and others. We also scanned *Flora Brasiliensis*, magazines such as the *Botanical Register* and *Curtis’s Botanical Magazine*, papers by Hallier, Urban, O’Donell, House, Matuda, Austin and many others. The online availability of a wide range of older journals through JSTOR and the Biodiversity Heritage library, as well as the help of library staff at University of Oxford and Royal Botanic Gardens, Kew, was invaluable. Further, the study of various herbarium specimens (see below) revealed putative types that we had not found in the literature search.

Today, the generation of this preliminary checklist could be much simplified by downloading data from a global repository, e.g., the *World Checklist of Vascular Plants* (Govaerts *et al.* 2021), Tropicos (Missouri Botanical Garden 2023), or World Flora Online (WFO 2023), a solution that was not available when we began our study. These databases make the compilation of a preliminary checklist a much easier task, especially in small or medium-size genera such as *Convolvulus* — although caution should be exercised when using them (Schellenberger Costa *et al.* 2023). Previously, in our study of *Convolvulus* (Wood *et al.* 2015b), we downloaded all *Convolvulus* names from the International Plant Names Index database (IPNI) as this allowed us to filter out a large number of long discarded names. In the case of *Ipomoea*, however, doing this would have implied handling over 3,000 names, many of them poorly known and thus adding an extra burden to this preliminary stage.

After the construction of the first draft of our list of names, these databases were nonetheless a useful resource to find names published more recently, and thus allowed us to keep our checklist regularly updated.

### Previous literature

Throughout history, botanical knowledge has been cumulative as well as corrective, and any taxonomic study inevitably builds on the work of earlier scientists going back at least to Linnaeus. In fact, making taxonomic decisions and assessing existing taxonomic literature of a group is accelerated and improved through experience, and thus the student taxonomist needs time to gain this type of experience. In the absence of experience, inexperienced researchers necessarily begin by searching for congruence between their own taxonomic decisions and those of previous taxonomists. This is a process of reciprocal illumination over time and is now greatly helped by molecular sequence data which enables the researcher to test new and prior taxonomic decisions that were based on morphology using an independent data source. Insights by previous botanists are also important for informing taxonomic decisions: it is pointless to reinvent the wheel as many species are correctly delimited, some relationships postulated with reasonable support, and many distinguishing characters have been highlighted for almost two hundred years.

Over time, new researchers accumulate experience of the taxonomic literature, taxonomists and taxonomic decisions, so learning which are more reliable than others. In the case of *Ipomoea*, for example, we found important nineteenth century (e.g., Choisy 1845; Meisner 1869) and twentieth century (e.g., Urban & Urban 1898; Standley 1937, 1938) works contain many errors. Serious questions are rightly asked about their taxonomic decisions. Others were more reliable — the taxonomic decisions of Carlos O’Donell, for example (e.g., O’Donell 1948, 1950); see Wood (2017) for an evaluation of *Ipomoea* taxonomists contribution and reliability.

Many species recognised by past authors still stand today and at least some of their genera and infrageneric groupings coincide with clades that have emerged from recent molecular studies. Thus, previous literature must be critically studied, diagnostic taxonomic characters analysed, and identification keys considered correct unless demonstrably mistaken.

It is also important to study original sources and to refer to protologues, type material, and species descriptions and notes in both academic and popular floristic works, the latter often with insights not reported in more academic publications. Such works might highlight diagnostic characters, particularly as colour and habit are often lost in herbarium specimens.

### Contacts & correspondence

Our work on *Ipomoea* benefited significantly from contacts with other botanists, travellers and local researchers interested in one or other aspect of *Ipomoea*, either by in-person and online meetings or through e-mail exchanges. Discussion and sometimes disagreements helped to clarify taxonomic characteristics, ecology, distribution, and variation. Time invested in correspondence and dealing with only marginally relevant queries was rarely wasted as contacts provided specimens, photographs, or other relevant information, as well as facilitated fieldwork and herbarium visits. Some contributed significantly to joint publications. An extensive list of our collaborators is provided in the ‘Acknowledgements’ section in Wood *et al.* (2020) and associated publications.

### Selection of specimens for study

Our research relied heavily on the use of herbarium specimens, an unparalleled resource for biodiversity studies. Herbarium specimens are invaluable as a permanent record of a species’ occurrence and characteristics, allow access to material collected all over the world in a short time, and facilitate taxonomic decision-making in different ways (Davis 2022). The study of herbarium specimens is thus central to any comprehensive taxonomic research. However, the number of herbarium specimens in any large plant genus is enormous (Bebber *et al.* 2010; Goodwin *et al.* 2015) and growing. As an example, at the time of writing, the GBIF database — which includes information only from a subset of the world’s herbaria — listed over 224,000 records of *Ipomoea* associated with herbarium specimens (GBIF Secretariat 2023), and we estimate the number of *Ipomoea* specimens worldwide exceeds 300,000.^2^ As it is clearly impossible to examine such a large number of specimens in a reasonable time-frame, it is important to prioritise what material is studied. The selection of herbaria to visit or request loans from is thus of paramount importance, especially in groups as diverse and widely distributed as *Ipomoea*.

To make informed decisions, it is important to know where a group is geographically most diverse. *Ipomoea*, for example, is an essentially tropical genus so there is little likelihood of finding a new species in a temperate region, even though its range extends to temperate areas. In our experience, previously collected putative new species are more likely to be found in herbaria with extensive collections from the priority areas (see *Index Herbariorum*), particularly those with large holdings and which have not been studied by previous experts. This includes both the main international herbaria but also national and regional herbaria with large collections, as well as herbaria where a previous expert had received collections for identification but did not publish the results. Efforts can be further focussed by homing in on regions where suitable habitats have been poorly collected.

In particular, in our work we prioritised seeing:

1. Type specimens of all species described in *Ipomoea*, transferred to *Ipomoea* from other genera, or considered as likely to belong to *Ipomoea*.
2. As many specimens as possible of rare species represented by < 10 collections, which make up a significant proportion of all accepted species (c. 49% in GBIF).
3. Specimens representing anomalous geographical or ecological records.
4. Representative specimens from all countries in the Americas (the main focus of our monograph) and all states in larger countries such as Brazil.

We made less effort to trace specimens of common, easily recognised species from other countries or states where they might be expected to occur. In line with this, two observations are important:

1. Many specimens were of poor quality and careful examination of these added little or nothing to our understanding of the morphology and variation of the species. However, such specimens might provide other kinds of valuable information — for example, significant range extensions.
2. Some thirty *Ipomoea* species accounted for more than half of all *Ipomoea* records in GBIF. In our experience, examination of more than 20 specimens of a given species is unlikely to add significantly to our understanding of morphological variation, and therefore little is gained by the detailed study of a large number of specimens of one species once material has been examined from its entire geographical range. This, of course, does not preclude a brief examination of all specimens consulted, as is required in any taxonomic study to check for misidentifications or other errors.

In addition to herbarium visits and specimen loans, the increasing availability of high-resolution images through virtual herbaria made our taxonomic work easier. In the case of *Ipomoea*, high-resolution images through JSTOR or the various virtual herbaria sites are almost as good as the herbarium specimens themselves, and important diagnostic information is usually visible (except for seeds, filament indumentum and stigma shape). Clearly, however, the image can only be as good as the original specimen. In our study, images were crucial in resolving some issues for accepted species,^3^ as well as issues involving species treated as synonyms.

### Field photographs and photographic records

In addition to herbarium specimens, we also had access to photographs of living plants taken by collaborators or by ourselves during field work. Photographs of living plants have the advantage over field observations of being permanently accessible and of being sourced from a variety of field workers. The disadvantage, of course, is that the taxonomist can only see what the photograph shows. However, in our case some photos supplied by contacts were of considerable importance,^4^ emphasising the value of collaborative contacts in the taxonomic process.

It was also important to take photographs of herbarium specimens we studied in person. This provided a record of specimens seen during our work in different herbaria, especially important when herbaria have not been digitised — as is the case with many Latin American collections. Importantly, we obtained photographs of almost all specimens from which we extracted DNA, which proved very useful if we needed to check the identity and morphology of a specimen if we did not have access to the specimen itself.

Both field photographs and images of specimens also play an important role in the public dissemination of our own results, publications both in print and online, and in the generation of identification tools.

### Field work

Although all descriptions and diagnoses in our study of *Ipomoea* were based essentially on the examination of herbarium specimens, we also saw over 25% of species found in the Americas in the field. We did field work in Argentina (2018), Bolivia (2012 to 2019 except 2015), Brazil (2014), Ecuador (2019), and Paraguay (2016, 2017 and 2018), resulting in new country records and the discovery of new species^5^ — these being added to the working checklist in the form of provisional names as they were discovered, as well as more precise ecological and phenological information. Although a few collectors are exemplary in the information they provide, in general, herbarium labels and literature provide very sparse ecological and phenological information.

Field work pre-dating and throughout the *Ipomoea* project (pre- and post-2012, respectively) has done much to enhance recognition of species and their ecology. It has drawn attention to characters that are diagnostic in distinguishing species. It has shown that non-morphological characters such as flowering season or habitat may help to characterise species, if and when accurately known. Fieldwork has shown how some characters (habit, indumentum, leaf shape and size, corolla colour and size) can be very variable within a species or, in the case of sepals, at different stages of development. Fieldwork has resulted in more samples of poorly known species or of poorly known parts of otherwise well-known species, such as their seeds and rootstock. It has provided information not available from herbarium specimens such as subtleties of flower colour (the dark centre in *I. australis* J.R.I.Wood & P.Muñoz, for example) and plant size and habit (the growth form of *I. juliagutierreziae* J.R.I.Wood & Scotland, the winged stems of *I. pterocaulis* J.R.I.Wood & L.V.Vasconc. — a character barely visible on the flowering shoots represented in herbaria). Field work also helped understanding infraspecific variation: seeing a range of specimens in different localities in the field (as in the herbaria) has made it possible to confirm whether a particular character (indumentum, for example) was constant in a population and over different populations, so confirming or rejecting taxonomic decisions.

## TAXONOMIC STUDY & WORKFLOW

### First steps in specimen re-identification

During the study of herbarium material, the previous identification on a label is often a clue to the right identification. In some cases, an earlier valid name simply needs to replace a well-known later synonym.^6^ In other cases, a wrong identification can be corrected because the name had been misapplied. In *Ipomoea*, for example, this was often the case in pairs of species that are morphologically similar.^7^ Of course, in the early stages of a taxonomic work it is almost impossible to know what is correctly named or not. Perhaps the best way to proceed is to secure a reference set of images of the types (through JSTOR, for example) or other reliably named specimens, and use these for comparison. The researcher should be cautious about accepting previous identifications which do not match well morphologically or geographically with their reference set. This is especially relevant for online data bases such as GBIF or Reflora, which should be used with great caution as many specimens are incorrectly identified (Goodwin *et al.* 2015, 2020).

Unidentified or incorrectly identified herbarium specimens can be sorted into putative species, which can then be examined for distinguishing characters. In this process, specimens can be sorted into separate ‘piles’ based on the presence of salient characteristics. Each pile can then be studied more intensively to identify additional diagnostic characters that confirm or reject the unity of the specimens grouped in individual piles. In our study, this process was mainly useful to sort unnamed material (i.e., specimens from the “indet” folders found in most herbaria) but was also useful in other situations, for example in distinguishing morphologically similar species.^8^

In the case of *Ipomoea,* and less so in *Convolvulus*, many species were only known from the type or a small number of collections. These may be problematic taxonomically as it is difficult to assess the reliability and consistency of characters. Herbarium visits, loans and specimen images made more specimens available, sometimes showing the existence of specimens with intermediate characters.^9^ In each case, a decision has to be made as to the taxonomic status of each species based on the information available, molecular and or morphological, backed sometimes by geography or ecology. Although we made decisions on the merits of each case, we tended to maintain species where information was limited as additional information may prove any changes premature.

Study of herbarium specimens, often but not only in the folders of unidentified material, often results in the discovery of new species (Bebber *et al.* 2010), which need to be added to the working checklist in the form of provisional names as they are discovered.

### Morphological character selection and taxonomic decisions

Although studies of morphological variation are always subject to a degree of informed subjectivity, in our experience some important, objective processes can be described. It is important to note that once specimens can be placed within a clade (see section C. Strategy for molecular sequence data), it is easier to delimit species because the specimens only need to be compared with a small number of other specimens and species from the same clade. Most clades in *Ipomoea*, for example, are relatively small in terms of species numbers, which facilitates species delimitation considerably.

Different morphological characters will be important in different degrees depending on the plant group studied, and therefore this section will necessarily focus on *Ipomoea* and *Convolvulus*. One such character in *Ipomoea* is the presence/absence of hairs on the exterior of the corolla, which appears nearly always to be constant in any given species. Even more significant is the range of variation seen in the sepals. They vary in size, relative size between inner and outer sepals, in indumentum, in ornamentation and texture and in the shape of the base and the apex. Sepal morphology is one of the most important diagnostic characters in *Ipomoea*.

The annual/perennial distinction is (apparently) diagnostic for individual species in *Ipomoea* but in most herbarium specimens this distinction can only be inferred with some uncertainty from the slender habit. However, there is a good proxy character: specimens that present both corollas and capsules (especially when both are frequent) are characteristically annual. In contrast, perennials are often marked by having corollas but no capsules or conversely capsules but no corollas. This is a partial explanation for why the fruit of many perennial species is unknown.

In the case of *Ipomoea*, we have observed that many characters seem to correlate closely, thus indicating that one or more can be inferred when another is present (and essentially constitute a single character, not separate characters). Thus, for example, a comose ovary correlates with a pubescent or comose capsule; a bilocular ovary with a 4-seeded capsule; a tri-globose stigma and a tri-locular ovary with a six-seeded capsule; and an erect habit with linear or oblong leaves and a terminal sub-racemose inflorescence. Although suboptimal, such correlations are a useful proxy during taxonomic work using herbarium specimens.

Other characters that are useful in distinguishing *Ipomoea* species, such as capsules, seeds, roots, flower colour or texture are not always practical when studying herbarium specimens as they were never collected or may have been lost in the drying process.

Finally, some species are delimited by what are essentially weak quantitative characters, based on differences in, e.g., the dimensions of the flower, leaf, or other morphological feature. These weak quantitative characters may be acceptable to delimit species if they are maintained over a large number of specimens. Conversely, we tended to reject them where an entity is represented by very few specimens or a single one. This underlines the importance of seeing a range of specimens to confirm the consistency of “quantitative” characters.

### Strategy for molecular sequence data

During our monographic work, molecular and morphological studies were conducted in parallel with constant feedback and reciprocal illumination. Our aim was to use DNA sequence data as an aid to taxonomic decisions, in contrast to traditional taxonomic studies where molecular data is often incorporated at a later stage to infer a phylogeny. Constant feedback and reciprocal illumination are the key aspects of our approach.

From the start of the project, our aim was to sequence as many specimens and species as possible, to test the monophyly of individual species whenever feasible. In addition, we also attempted to sequence specimens deemed interesting based on their morphology or their provenance. However, DNA sequencing and data analysis can be very time-consuming and should be carefully planned, particularly when working within the constraints of a limited budget.

With this in mind, we decided to use DNA barcodes (*nrITS*, *matK*) as primary molecular markers for our taxonomic studies. In the case of *Ipomoea*, these DNA regions had been shown to provide acceptable levels of phylogenetic resolution (Miller *et al.* 1999; Manos *et al.* 2001), and we later confirmed that species monophyly and placement within the main clades is generally consistent with more recent phylogenies using genomic-scale data (Muñoz-Rodríguez *et al.* 2019). However, the limitations of DNA barcodes for phylogenetic studies are well known and *Ipomoea* is no exception: DNA barcodes alone are often not enough to resolve the monophyly of all species or the relationships between closely related species. Nevertheless, once these limitations are acknowledged, DNA barcodes are still a useful, fast, and affordable way to incorporate DNA sequence data in taxonomic studies — especially when they are supported by genomic backbone phylogenies (see below). Briefly, DNA barcodes allow a quick, rough-and-ready placement of individual specimens in a phylogenetic context, allowing the rapid detection of close relatives of any given species, the putative monophyly of individual species, the detection of clearly misidentified material and the placement of limited material that is poorly preserved. This reduces the number of species with which morphological comparisons need to be done, overall accelerating comparative studies.

It must be noted that we made no decisions solely based on DNA barcode data, especially as we were aware of the limitations of using DNA barcodes. Instead, we always combined DNA data with morphological observations, often going back to studying herbarium collections in the light of new molecular results. This constant integration between molecular and morphological data throughout the taxonomic workflow results in a more complete taxonomic knowledge and better-defined species boundaries.

As part of this project, we generated DNA barcodes for around 65% of *Ipomoea* species, and we continue to generate them on a regular basis (Jara *et al.* 2020; Wood *et al.* 2024). We attempted to sequence as many species as possible, and several accessions per species whenever available. This was especially important for widespread species or species exhibiting marked morphological variability. As a result, and although it was not our primary objective, we soon amassed an extensive amount of data that allowed us to investigate other questions beyond species-level taxonomy, for example the relationship between *Ipomoea* and other genera in the tribe Ipomoeeae (Muñoz-Rodríguez *et al.* 2019; Muñoz‐Rodríguez *et al.* 2023b). However, as we progressed in our study, we realised DNA barcodes would not be enough to answer some of the most interesting evolutionary questions that arose, including the relationship between sweet potato (*I. batatas* (L.) Lam.) and its close relatives. These relationships and those in other recently originated clades in *Ipomoea* (Carruthers *et al.* 2020a) are largely resolved as polytomies in *nrITS* and *matK* phylogenies. Thus, two years into the project we decided to obtain genomic-scale sequence data for a subset of species (c. 211 species, 25% of total species in tribe Ipomoeeae) representative of the diversity existing within the genus. Our aim was four-fold: 1) to generate more robust molecular phylogenies and corroborate the results of DNA barcode phylogenies; 2) to confirm the relationship between the main clades in *Ipomoea* and with other genera in Ipomoeeae; 3) to test congruence between nuclear and plastid data; and 4) to investigate the relationship between the sweet potato and its close relatives in depth. We used Hyb-Seq (target enrichment of low copy nuclear regions plus genome skimming to capture the chloroplast and highly repetitive nuclear regions), which was at that point a newly developed approach (Weitemier *et al.* 2014) and sequenced four 96-well plates. This method was cost-effective and allowed us to assemble hundreds of low copy nuclear DNA regions and whole chloroplast genomes. We sequenced one specimen per species for most species, several specimens for some species to test monophyly of individual species, and multiple specimens per species for the sweet potato and its close relatives to test species limits in the Batatas clade.

This two-tier sequencing strategy combining DNA barcodes and Hyb-Seq proved very successful. First, we confirmed that our DNA barcode phylogenies were largely congruent with the genomic phylogenies, providing further support for their use in the taxonomic decision-making process. Secondly, we confirmed that all smaller genera in Ipomoeeae were nested within *Ipomoea* — and synonymised them (Muñoz-Rodríguez *et al.* 2019). Thirdly, we revealed the existence of two rapid evolutionary radiations in the American continent (Muñoz-Rodríguez *et al.* 2019; Carruthers *et al.* 2020a) and the prevalence of other evolutionary patterns in the genus, such as long-distance dispersal (Muñoz-Rodríguez 2019). Finally, we obtained important insights into the origin and evolution of the crop species (Muñoz-Rodríguez *et al.* 2018, 2022a).

### A DNA barcode phylogeny as another taxonomic character

The key part of our methodology was the integration of a DNA barcode phylogeny (*nrITS* in the case of *Ipomoea*) in the taxonomic process. In essence, we used DNA barcode phylogenies as an additional taxonomic character alongside morphological characters and ecological and geographical data from field observations, literature, and specimen labels. As explained above, the use of a DNA barcode phylogeny facilitates the quick identification of the correct evolutionary context (clade) of a given specimen so reducing the number of species with which the specimen needs to be compared and facilitating subsequent morphological comparison (Williams *et al.* 2014). In this context, it was particularly useful to have the universal approach of a monograph rather than the country-based approach of a flora.

For many species, initial species delimitation based on morphology were corroborated by multiple accessions of the species forming a highly supported clade in the barcode tree. In such cases, the delimitation of the species was clearly consistent with both morphology and molecular data. In contrast, if specimens a priori assigned to the same species were distantly related in the barcode phylogeny — strong disagreement between a morphology-based hypothesis and molecular data — it was necessary to re-examine all data to check whether the conflict was real (i.e., different specimens represented different evolutionary entities that had not been identified in morphological studies) or simply, in many cases, because they were wrongly identified.

Thus, in those cases where morphological observations recognised two distinct species, we accepted them as distinct if either of them was more closely related to any other species in the phylogenetic tree. Similarly, if our morphology-based observations led to a hypothesis that two previously recognised species could in fact be conspecific, we used *nrITS* as evidence corroborating this if the specimens from both species formed a clade albeit unresolved or with accessions from both species intermingled.

It is important to note that we were extremely cautious interpreting the results of the DNA barcode phylogenies when species hypotheses based on morphology were not corroborated by *nrITS*. This caution reflects the well-known limitations associated with *nrITS* phylogenies mentioned above (short DNA sequences, lack of resolution, low support values, paralogy). Therefore, when we had a species hypothesis based on morphology but not corroborated by *nrITS* due to lack of resolution, we accepted our morphology-based hypotheses, as we could not be sure whether the phylogeny reflected an accurate pattern or the pattern observed was simply due to the limitations associated with *nrITS*. We adopted this approach based on our experience with the group of species closely related to the sweet potato (Muñoz-Rodríguez *et al.* 2018). In the *nrITS* phylogeny, most species in this group were unresolved in a large polytomy due to lack of sequence variation but were resolved as monophyletic with more nuclear and chloroplast sequence data.

### Species narratives: how taxonomic decisions are made

There is a tendency by researchers to see a taxonomic revision or monograph as a done deal, like the solution to a mathematical problem, rather than like a historical or literary revision which is always open to reinterpretation. It is surprising how few taxonomists ever publish a revision or correction of their work even when going on to study the same group for years after their original work was published, and when numerous flaws or omissions are apparent. Further collections and new methods as well as reinterpretations always mean that no taxonomic monograph is ever final.

Students will inevitably have difficulty in deciding what to accept and what to reject. An experienced practitioner will notice anomalies, whether morphological, phylogenetic, geographical or ecological. Two specimens apparently the same but from different geographical regions or very different habitats raise suspicions and should be investigated. Similarly, two specimens of apparently the same species which are resolved differently through molecular study suggest a sampling error, a cryptic species or the need to re-evaluate morphological differences.

Taxonomic literature often lacks specific examples and enough narrative detail to determine how taxonomic decisions are actually made. Although this may seem obvious to practising taxonomists, it may not be so for researchers in other disciplines or a broader audience. In the supplementary information to Muñoz-Rodríguez *et al.* (2019), we provided eight specific examples of how we made taxonomic decisions. We think those examples were useful for four main reasons. First, they highlight the “heuristic” process of species delimitation (Wells *et al.* 2021). Second, they demonstrate the cumulative element in species delimitation as new material, photographs and observations become available over time. Third, they show how new collections often have to be compared with inadequate material and partially known existing species. Fourth, they provide practical examples of how we used DNA barcode phylogenies, and show how a carefully constructed phylogeny, albeit based on a single DNA marker, can provide clarity and subsequently play an important role in making or at least facilitating taxonomic decisions.

These examples, however, had to be published as part of a 50-page supplementary information document and went largely unnoticed. Thus, we here present four examples extracted from this supplementary material, and we direct the reader to the original document for additional examples (Muñoz-Rodríguez *et al.* 2019, Supplementary Material). We also indicate why we consider these examples important in the context of our work. Although there were other contributors, much of the specimen-based work was carried out by John R. I. Wood, one of the authors of this paper, and therefore the examples are written in the first-person singular.

## EXAMPLE 1

This example shows how the integration of morphological and molecular data enable the discovery of new species. This is one of several cases in our work on *Ipomoea*.

*Ipomoea longibarbis* J.R.I.Wood & Scotland is a species of the Bolivian Chaco (Wood *et al.* 2015a). It was first collected in 1952 by Martín Cárdenas but languished unrecognised in the Lillo herbarium (LIL, Argentina) until 2014. The same species was collected a couple of times in the 1990s and then quite frequently after 2000, always along the fringes of the western Chaco in Bolivia.

I became aware of this plant around 2010, when examining material in the Santa Cruz herbarium (USZ, Bolivia) at the start of our focus on *Ipomoea*. My first impression was of a species closely related to *Ipomoea argentinica* Peter, which is common around Santa Cruz, but which is immediately distinguished by the conspicuous spreading hairs on the calyx as well as the deciduous bracteoles. Although I had never collected this species myself, I was able to borrow specimens from USZ to study in the UK.

In 2012, I was able to show this material to Rosangela Simao-Bianchini from the Instituto de Botânica de Sao Paulo (Brazil) during her visit to Kew. She suggested the plant was *Ipomoea rubens* Choisy. On comparing the specimens, it was easy to see why she had suggested this, as the sepals are almost indistinguishable. I was, however, unconvinced, as I knew *I. rubens* to be a plant of river and lake margins, whereas the Chaco plant was a plant of dry forest. There were also some slight morphological differences, *I. rubens* often having shallowly 3-lobed leaves while those of the Chaco species were always entire. Closer study showed that the hairs on the seeds of the Chaco species were long, fine and caducous while those of *I. rubens* were shortly but densely pilose and persistent.

In this situation, I felt that seeing the Chaco plant in the field and collecting additional material was a priority. In early 2013 we made a field trip to Bolivia, found the plant and brought specimens back to Oxford for study. These were sequenced along with specimens of *Ipomoea rubens* from Bolivia and elsewhere. The molecular analysis showed that the two species were not closely related, *I. rubens* belonging to the essentially Old World clade of *Ipomoea* while the Chaco species was part of a radiation in the Carnea Clade (or Clade A; see Muñoz-Rodríguez *et al.* 2019, 2023b). During 2013 – 14, additional material of the Chaco species was found in various herbaria in Argentina and the United States as well as in Bolivia, totalling 14 collections. We described it as a new species with the name *Ipomoea longibarbis* (Wood *et al.* 2015a).

At least six specimens of *Ipomoea rubens* were sequenced for *nrITS*, three from the New World and three from the Old World. These showed the species to be monophyletic and strongly suggested an African origin, as it belongs to a well-supported clade of wholly African species. It is not clear how *I. rubens* arrived in the New World, but it is locally abundant and has every appearance of being a native species in Bolivia, Argentina, Paraguay, and Brazil. *Ipomoea rubens* and *I. longibarbis* are good examples of convergence of traits and of the importance of different ecologies in suggesting taxonomic distinction.

## EXAMPLE 2

This example shows how morphological examination provided the clue to the discovery of a new species that was then confirmed by molecular analyses.

*Ipomoea aristolochiifolia* G.Don is one of the more widespread species in the American continent, extending from Argentina to northern Mexico. It is a slender species with a relatively small blue corolla with a pale tube, subequal, often warted, narrowly ovate sepals with a white margin and a relatively large, often rostrate capsule. Although it is very variable in some characters such as indumentum and leaf dentation, it is usually easily distinguished by an unusual character in *Ipomoea*: the peduncle passes through the leaf sinus.

In 2014, I visited the La Paz herbarium (LPB) and was shown specimens of what appeared to be a rather large-flowered hairy form of *Ipomoea aristolochiifolia*. It transpired that there were two separate collections made on successive days from the same area. I requested duplicates of these specimens to take back to Oxford together with images of the collection showing the distinctive peduncle passing through the sinus of the leaf base. I examined these carefully and suspected they represented a distinct species because of the larger dimensions of all flower parts and leaves as well as the denser indumentum of all vegetative parts. In Oxford, samples of this species were sequenced and came up with a slightly surprising result. It showed that it was indeed in the same clade as *Ipomoea aristolochiifolia* but was more closely related to another putative new species from further south in Bolivia, which did not have the distinctive peduncle character. We described both these species new to science as *I. huayllae* J.R.I.Wood & Scotland and *I. odontophylla* J.R.I.Wood & Scotland in *Kew Bulletin* (Wood *et al.* 2015a). Both are essentially pin-point endemics but fortunately grow in protected areas.

## EXAMPLE 3

This example shows how the study of herbarium specimens and the extensive query of past literature during our monographic study led to the resurrection of a forgotten name.

*Ipomoea montecristina* Hadač was collected by Hadač on Monte Cristi in Guantanamo province (in Eastern Cuba) in 1968. Hadač published it two years later (Hadač 1970), but the types were not deposited in a Cuban herbarium, and it remained unnoticed by Cuban botanists and by the international botanical community for many years. The species was not recognised by the botanical community (e.g., Austin & Huáman 1996) nor by me when I visited Cuba in November 2015.

I began my visit to Cuba at the Instituto de Ecología y Sistemática (HAC), where I examined all collections of *Ipomoea*. Amongst these were two collections from Eastern Cuba, which appeared to represent a new species. The specimens lacked corollas but the sericeous leaves and coriaceous sepals were distinctive. Later during my trip, I visited the herbarium of the National Botanical Garden (HAJB) and found around ten collections of the same species. All of these originated from Eastern Cuba, principally from limestone “pinares” (pine forests) at Monte Cristi and Abra de Mariana near San Antonio del Sur. These comprised better material with corollas, capsules, and seeds. None had been identified by Dr Manitz from Berlin (a specialist in the *Ipomoea* of Cuba) and I was confident they represented an undescribed species and annotated them as such.

Soon after my return to Britain, when I was revising the *Catalogue of Seed Plants of the West Indies* (Acevedo-Rodríguez & Strong 2012) and checking whether my list of Caribbean *Ipomoea* species was complete, I saw *I. montecristina* Hadač listed. The epithet immediately suggested the plant I had seen in the Cuban herbaria, and reference to the protologue confirmed this suspicion. The description and type locality indicated clearly that the Cuban plant was indeed *I. montecristina.* This led me not to describe the species as new, but to resurrect this name and publish the full description of this forgotten Cuban species.

## EXAMPLE 4

This example shows how we combined evidence from morphological and molecular data to synonymise two species previously thought to be distinct.

*Ipomoea acanthocarpa* (Choisy) Aschers. & Schweinf. was first discovered by the Austrian collector Teodor Kotschy in Sudan in 1839. It was distributed by Hochstetter under this name to various herbaria but was only formally published as a new species in 1845 under the name *Calonyction acanthocarpum* Choisy (Choisy 1845). Why Choisy placed it in the genus *Calonyction* Choisy is unclear, as he had based this genus on having a conspicuous corolla similar to *Datura* and with exserted stamens. This does not fit *Ipomoea acanthocarpa* at all, but Choisy could not have known this as he did not describe the corolla. Just over 20 years later, Ascherson and Schweinfurth transferred the species into *Ipomoea* (Schweinfurth 1867).

*Ipomoea acanthocarpa* is widespread across the Sahel region of Africa, extending from Senegal in the west to Sudan and Ethiopia in the east. Collections are not very numerous, especially in the east of its range, but it is present in most countries in this extensive region.

Over a hundred years after its publication, during the course of his revisionary work on American Convolvulaceae, O’Donell came across a specimen of an unnamed *Ipomoea* from Peru (*Haught* 142) and another one from the other side of the continent in eastern Brazil (*Froes & Black* 24742). He correctly identified these as the same species describing them under the name *Ipomoea piurensis* O’Donell in 1953, after the Peruvian department of Piura. Over the years, *I. piurensis* has been reported from a number of American countries including Colombia, Ecuador, Guyana, and Venezuela (Austin & Huáman 1996) but never in great frequency.

No one had considered *Ipomoea acanthocarpa* and *I. piurensis* could be conspecific before we sequenced examples of the two species during the course of our studies. We sequenced in total eight representative samples from Africa and the Americas, and the two species formed a well-supported clade with the samples intermingled. Examination of a large number of African and American specimens in different herbaria indicated that there were no morphological differences and thus the two species should be treated as the same under the oldest name, *I. acanthocarpa*. In passing, it is worth noting that this species is widespread but very dispersed in its distribution in both continents. Recent collections have added Costa Rica, Colombia, and Bolivia to its American distribution, the last two as a result of our study.

The molecular sequences revealed something else interesting. Although *Ipomoea acanthocarpa* was first found in Africa in 1839, it clearly belongs to a larger clade of entirely American species including *I. bahiensis* Willd., *I. squamosa* Choisy and *I. eriocalyx* (Mart. ex Choisy) Meisn. This suggests that it is a plant of American origin that has colonised Africa, going in the reverse direction to, for example, *I. rubens*. Whether the introduction to Africa was by ancient long-distance dispersal or by relatively recent human activity is unclear but its wide distribution in Africa might suggest the former.

### Publication

When the *Ipomoea* monograph finally came out in 2020, 423 species were fully described, and nomenclatural and typification issues were resolved in hundreds of cases. The monograph included photographs, identification keys for geographical areas and well-defined groups and 257 original illustrations (Wood *et al.* 2020). We considered it important to include full illustrations of as many species as possible in the monograph, because an image adds a further dimension to a written description. This meant additional costs incurred and time invested by us and by artists both in-house and in other countries. Illustrations and images associated with a monograph have to be carefully planned as they are an important element of the published work. We published the phylogenies generated in the project in a separate paper addressing several evolutionary questions and the classification of the tribe Ipomoeeae (Muñoz-Rodríguez *et al.* 2019). Although not formally a part of the monograph, this associated work was obviously essential to the successful completion of the monograph.

Finally, we had to decide where and how to publish the monograph. This is an important question, especially in the current context of scientific publication and metrics. In the case of our *Ipomoea* and *Convolvulus* monographs, we considered it important to make them available open access in order to reach the widest audience possible, especially as the geographical distribution of *Ipomoea* coincides with countries rich in biodiversity but often with limited access to publications. However, researchers cannot overlook open-access publishing costs. Our initial research funding, for instance, did not cover these costs, so it was important that the considerable open access charge (€ 8500) could be covered by the block open access fund held by the University of Oxford for science funded by the UK Research Councils. This required publishing the monograph in a recognised peer-reviewed journal (i.e., not as a book), in our case *PhytoKeys* (Wood *et al.* 2020), and the article is treated as a research article not a book in all respects — although it is the only article in its issue. Given the size of the monograph and the amount of information, the review process and editorial work extended over a year.

## AFTER PUBLICATION: IMPACT AND NEW STUDIES ENABLED BY THE MONOGRAPH

The completion of a taxonomic monograph is often seen as the end of a long, gruelling journey: the task is completed, the team can disperse, and the author(s) move on to fresh fields of research. In our experience, however, it would be better seen as only a milestone along a journey undertaken to understand the complexities of a group of plants. A monograph is not the end of a journey, but a step forward enabling subsequent research to be based on a more robust taxonomic framework. In our case, the team has continued to work together and funding, at least for sweet potato studies, has continued.

The publication of the monograph early in 2020 (Wood *et al.* 2020) constituted an important milestone but it is informative to look at some of the things that happened afterwards and publications that have been completed since. It is also worth noting that, at the time of revising this paper, we learned that the monograph was one of the three most cited papers published in *PhytoKeys* in 2020, with 25,000 accumulated views. This we think reflects the potency of a global taxonomic treatment.

### New species and new records

Already before the publication of the monograph, during the review process, we became aware of the existence of one putative new species endemic to Peru (Species D, number 425, in the monograph, Wood *et al.* 2020). This species was soon published as *Ipomoea noemana* E.Jara, P.Muñoz & H.Beltrán (Jara *et al.* 2020), and subsequent fieldwork between 2021 and 2023 has identified several additional populations along the Marañón river valley (Enoc Jara and Paúl Gonzales, *pers. comm.*, and our own observations) which we are now evaluating.

In addition, recent fieldwork in Ecuador has confirmed the existence of four additional new species in the country and has clarified the delimitation of several species — including the re-establishment of *I. ophiodes* Standl. & Steyerm., which we wrongly treated as a synonym of *I. regnellii* Meisn. in the monograph (Wood *et al.* 2024).

Two other new species, one from Brazil — *I. lanifolia* D.Santos & Buril (Santos *et al.* 2021) — and one from Ecuador — *I. aequatoriensis* T.Wells & P.Muñoz (Muñoz-Rodríguez *et al.* 2022a) — have also been recently published. Given its importance, the discovery of *I. aequatoriensis* is discussed in the Sweet potato origin section.

Although the absence of additional new species in other American countries suggests our monograph provided good coverage, it is likely that more will appear, especially through the study of other local herbaria in tropical countries or additional fieldwork in poorly collected areas.

In parallel, correspondents and collaborators have confirmed new country records of *Ipomoea rosea* Choisy from Bolivia and *I. discolor* (Kunth) G.Don from Colombia, while a visit to Colombia in 2022 by John Wood revealed several additional new country records and a putative new subspecies of *I. schomburgkii* Choisy. Fieldwork by colleagues in Bolivia in 2022 has also highlighted the presence of significant variation in *I. lilloana* O’Donell and *I. cheirophylla* O’Donell.

### The phylogeny and its implications

The phylogenies that we produced (*nrITS*, target enrichment, whole chloroplast genomes) facilitated several subsequent studies. First, they helped us confirm that all smaller genera in the tribe Ipomoeeae were nested inside a paraphyletic *Ipomoea* (Manos *et al.* 2001; Stefanovic *et al.* 2002, 2003; Muñoz-Rodríguez 2019). We thus proposed to recognise an expanded *Ipomoea* as the only genus in Ipomoeeae (Muñoz-Rodríguez *et al.* 2019). Although species continued to be published in one of the segregate genera, *Argyreia* (Lawand & Shimpale 2021; Rattanakrajang *et al.* 2022), no logical rationale for the rejection of our proposal has been made. We discussed this proposal extensively in the original paper and in two additional publications (Muñoz-Rodríguez *et al.* 2022b; Muñoz‐Rodríguez et al*.* 2023b).

Our phylogenies also left issues unresolved. These included the existence of potential polyphyletic species and extensive polytomies, notably in Clade A1 (the Carnea clade sensu Muñoz-Rodríguez *et al. *2023b). We are currently working to clarify some of these issues, including some related to widespread Old World species (e.g., *Ipomoea biflora* L., *I. triflora* Forssk.). Work to clarify the origin and extent of the two potential rapid radiations in the group (Carruthers 2019; Muñoz-Rodríguez *et al.* 2019; Carruthers *et al.* 2020a) also continues, making use of high-throughput sequencing.

### Time-calibrated phylogenies and diversification studies

After conducting fieldwork in Paraguay and Bolivia, Tom Carruthers investigated the timing of evolutionary diversification in *Ipomoea*. This had a theoretical focus, namely characterising the types of questions that can be addressed about plant evolution arising from a comprehensive taxonomic and phylogenetic framework (Carruthers *et al.* 2020b; Carruthers & Scotland 2020, 2021a, 2021b, 2022, 2023), with a focus on specific questions related to *Ipomoea.* The taxonomic work was, in fact, central to illuminating specific questions about the evolution of *Ipomoea* and offered a potential for truly original macroevolutionary insights. The combination of a robust taxonomic framework and carefully planned diversification studies resulted in a better understanding of when the edible storage root of the sweet potato evolved, and the characterisation of explosive rates of evolutionary diversification in neotropical *Ipomoea*.

Although taxonomy and phylogenetics can clarify the evolutionary relationships between species, they are not directly informative about timescales. To address questions about the macroevolution of *Ipomoea*, we took additional methodological steps that included divergence time estimation (placing the branching events in a phylogeny on an absolute timescale) and diversification rate estimation (estimating the absolute rate of diversification in different lineages). Importantly, the comprehensive taxonomic framework on which the project rested enabled us to clarify the power and effectiveness of these additional approaches and to identify limitations that are intrinsic to them in a context that is not undermined by a poor or incomplete taxonomic framework. We investigated how, regardless of the number of genetic loci analysed, species sampled, robustness of estimated evolutionary relationships or methodological approach followed, molecular substitution rate variation that acts consistently across entire genomes (a common pattern within *Ipomoea*) leads to significant error when estimating divergence times. Likewise, shifts in diversification rates caused additional problems in diversification rate and divergence time estimation, this issue actually becoming more acute with increased taxon sampling. Meanwhile, we also investigated how common assumptions about the temporal signal in the fossil record — incorporation of which is necessary for divergence time estimation — cause highly erroneous divergence time estimates. This issue was especially acute in *Ipomoea*, after the discovery of the Solanaceae fossil *Physalis infinemundi* Wilf (Wilf *et al.* 2017) that led to dramatic changes in the understanding of when *Ipomoea* evolved (Convolvulaceae is part of order Solanales). Overall, this set of analyses highlighted the extensive, often underappreciated uncertainties that underpin macroevolutionary inferences. We directly incorporated these issues into subsequent publications addressing evolutionary questions within *Ipomoea* and sweet potato (Carruthers 2019; Muñoz-Rodríguez *et al.* 2019; Carruthers *et al.* 2020a) and also a number of theoretical issues underpinning divergence time analysis (Carruthers *et al.* 2020b; Carruthers & Scotland 2020, 2021a, 2021b, 2022).

In terms of the macroevolution of *Ipomoea*, our initial focus was on the timing of the origin of the most economically important species in the genus, *I. batatas*. The project had resolved the taxonomy and phylogenetic relationships of *I. batatas* and its close relatives, confirming that *I. batatas* is the only species among its closest relatives to possess an edible storage root. We therefore used this framework to estimate when the edible storage root of *I. batatas* evolved, and specifically, whether it evolved prior to a likely human domestication. By biasing divergence time estimates to as young an age as possible and showing that even in this situation *I. batatas* diverged from its closest relative at least a hundred thousand years ago, we demonstrated that the origin of the storage root in sweet potato pre-dated human domestication. Alongside this, we performed a broader scale analysis of the timing of evolutionary diversification in *Ipomoea*. Despite the intrinsic problems underpinning divergence time estimation that we had analysed extensively, and the inevitable uncertainties that this created, we were able to confidently infer explosive rates of diversification among neotropical species of *Ipomoea*, these being some of the highest recorded within the plant kingdom (e.g., Baldwin & Sanderson 1998; Hughes & Eastwood 2006; Givnish *et al.* 2009; Koenen *et al.* 2013).

### Sweet potato origin

Our monographic work facilitated extensive research on the origin and evolution of the sweet potato, including the identification of sweet potato’s closest diploid relative, *Ipomoea trifida* (Kunth) G.Don. (Muñoz-Rodríguez *et al.* 2018, 2019). Perhaps the most important issues left unresolved, however, related to wild populations of the sweet potato that might include a possible ancestor of the cultivated plant. By the time we published the monograph, we had identified populations in coastal Ecuador as the most promising to search for this ancestor (Muñoz-Rodríguez *et al.* 2022a). The initial observation that led to this discovery lay in the shape of the calyx in these specimens compared with sweet potato and *Ipomoea trifida* (Kunth) G.Don, a minor difference only noticed after a substantial period of comparative study (Muñoz-Rodríguez *et al.* 2022a). Phylogenetic studies showed that these Ecuadorian populations previously identified as sweet potato, in fact, represented a distinct species, *Ipomoea aequatoriensis*, most likely a direct descendent of the progenitor species of sweet potato. This new species was formally described in 2022 (Muñoz-Rodríguez *et al.* 2022a), and further field work and herbarium visits in 2022 revealed that *I. aequatoriensis* is, in fact, widespread in the country and possibly also present in Colombia and northern Peru (Wood *et al.* 2024).

### Conceptual questions

We have also had the opportunity to focus on theoretical aspects of systematics research that are often overlooked. First, we analysed how long it takes to accumulate a minimum amount of information of a plant species to enable subsequent studies (we found it takes, on average, seventy years to ‘discover’ a species, i.e., to obtain as little as 15 correctly identified specimens; Goodwin *et al.* 2020). We also published two theoretical papers on the concept of species as seen by taxonomists (see below), as well as another paper and a book chapter on Angiosperm classification and the reconciliation of monophyly, diagnosability, completeness and nomenclatural stability in modern systematics (Muñoz-Rodríguez *et al.* 2022b, 2023b).

A central problem in systematics remains the difficulty of resolving the tension between species delimitation and the process of evolution. Repeated recognition of this “species problem” in the course of our intensive monographic study of *Convolvulus* and *Ipomoea* led to the initial formation of the notion of a “species as a heuristic” concept. We introduced this idea as follows: “Our view is that species and species delimitation can be viewed as a heuristic allowing an approach to problem solving or discovery that employs a practical method not guaranteed to be optimal or perfect, but sufficient for the immediate goals” (Wood *et al.* 2020). This was then further developed in two subsequent publications exploring how heuristics relate to both theoretical ideas about speciation, and practical methods of delimitation (Wells *et al.* 2021), and how different forms of systematic data should be integrated within a heuristic approach to species delimitation (Wells *et al.* 2022).

### Additional publications and collaborations

Other spin-offs from our monographic research on *Ipomoea* and the origin of the sweet potato include a book chapter on its presence and arrival on Easter Island (Muñoz-Rodríguez *et al.* 2022c) and the publication of an invited contribution in the *Indian Journal of Economic and Taxonomic Botany* revising the state of knowledge of Convolvulaceae in the country and highlighting important aspects for future research (Wood *et al.* 2022). We have also established wider links and we have served in a consultative role in floristic accounts in southern Africa (Wadley *et al.* 2021), New Guinea (Cámara-Leret *et al.* 2020), Mexico (Deloya Brito *et al.* 2023), and sweet potato wild relatives conservation (Khoury *et al.* 2015), amongst others.

## CONCLUSIONS

Botanical monographs play a pivotal role in taxonomic research and are a unique contribution to our understanding of the world’s biodiversity. When conducted at a global scale, taxonomic monographs also provide important information on the evolution of a group of species, its biogeography, and many other questions. In this article, we have summarised a decade of monographic studies on *Convolvulus* and, especially, *Ipomoea*. Our work has been used and cited widely, resulting in a wider reach beyond our own research agenda (e.g., Iriarte *et al.* 2020; Capriles *et al.* 2022; Mayo 2022). This is especially rewarding as it shows the impact of a taxonomic monograph in the wider research ecosystem, which often fails to recognise how important good taxonomic knowledge is for the study, understanding, and protection of the world’s biodiversity.

## Footnotes

1 See (Muñoz-Rodríguez *et al.* 2019, 2023b) for an update on this question.

2 In total, for the monograph we studied physical specimens from 47 European and American herbaria (Wood *et al.* 2020), alongside an extensive range of images available online. These images allowed us to study material from other herbaria and to locate and confirm the identity of almost all types. We only rarely needed to request images of types, but those provided by G, LE, MA, TO and W (acronyms according to Thiers 2018) were especially useful. We requested the loan of type specimens in very few cases.

3 For example around *Ipomoea peruviana* O’Donell and *I. cuscoensis* J.R.I.Wood & P.Muñoz; *I. longeramosa* Choisy and *I. dajabonensis* Alain; or *I. carmesina* Proctor and *I. lineolata* Urb.

4 Photos for example of *Ipomoea platensis* Ker Gawl. or *I. retropilosa* subsp. *cundinamarcana* J.R.I.Wood & Scotland.

5 It is difficult to determine how many new species have been discovered through field work. By field work by ourselves or collaborators, and nothing else — i.e., no herbarium specimens—, just a few (*Ipomoea appendiculata* J.R.I.Wood & Scotland, *I. chiquitensis* J.R.I.Wood & Scotland, *I. noemana* E.Jara, P.Muñoz & H.Beltrán or *I. prolifera* J.R.I.Wood & Scotland, for example). However, field work combined with herbarium studies have revealed numerous species, essentially cross-feeding each other.

6 Some examples in our work: *Ipomoea alba* L. (published in 1753) replaced *I. bona-nox* L. (published in 1762), and *I. indica* (Burm.) Merr. (published in 1755 as *Convolvulus indicus*) replaced *I. congesta* R.Br. and *I. acuminata* (Vahl) Roem. & Schult. (published in 1810 and 1819 respectively).

7 The widespread species *I. hederifolia* L., for instance, has been frequently misidentified as *I. coccinea* L., which has been shown to be a localised endemic in Eastern United States. Similarly, the widespread *I. mauritiana* Jacq. has frequently been mislabelled as *I. digitata* L., a Caribbean species endemic to Hispaniola (Haiti and Dominican Republic).

8 Example: *I. eremnobrocha* D.F.Austin from *I. isthmia* J.R.I.Wood & Buril.

9 Many examples could be cited, including *I. amnicola* Morong and *I. chiliantha* Hallier f.; *I. tubulata* Sessé & Moc. and *I. dumosa* (Benth.) L.O.Williams; *I. reticulata* O’Donell and *I. saopaulista* O’Donell; *I. campestris* Meisn. and *I. stenophylla* Meisn.; *I. grandifolia* (Dammer) O’Donell, *I. cynanchifolia* Meisn. and *I. ramosissima* (Poir.) Choisy; *I. alterniflora* Griseb., *I. obtusata* Griseb. and *I. excisa* Urb.; *I. lenis* House and *I. durangensis* House.
